# Genome-wide selection reveals candidate genes associated with multiple teats in Hu sheep

**DOI:** 10.1080/10495398.2024.2380766

**Published:** 2024-07-21

**Authors:** Wen Zhou, Cheng-long Zhang, Zhipeng Han, Xiaopeng Li, Xinyu Bai, Jieru Wang, Ruizhi Yang, Shudong Liu

**Affiliations:** College of Animal Science and Technology, Tarim University, Xinjiang, China

**Keywords:** Hu sheep, multiple teats, candidate genes, selection signals

## Abstract

Increasing the number of teats in sheep helps to improve the survival rate of sheep lambs after birth. In order to analyze the candidate genes related to the formation of multiple teats in Hu sheep, the present study was conducted to investigate the genetic pattern of multiple teats in Hu sheep. In this study, based on genome-wide data from 157 Hu sheep, Fst, xp-EHH, Pi and iHS signaling were performed, and the top 5% signal regions of each analyzed result were annotated based on the Oar_v4.0 for sheep. The results show that a total of 142 SNP loci were selected. We found that *PTPRG, TMEM117* and *LRP1B* genes were closely associated with polypodium formation in Hu sheep, in addition, among the candidate genes related to polypodium we found genes such as *TMEM117, SLC25A21* and *NCKAP5* related to milk traits. The present study screened out candidate genes for the formation of multiple teats at the genomic level in Hu sheep.

## Introduction

Sheep is an important breed of livestock After thousands of years of domestication, artificial selection has led to the diversity of sheep populations, the Hu sheep is China’s famous Taihu breed, with high fertility, year-round estrus, its lactation function is excellent, and its nutritional value is worth studying, Chen et al. found that the Hu sheep’s milk contains a unique lactic acid bacterium, which has vertical transmission advantages.^1^ Traditionally, the greater the number of teats in a mammal, the greater the number of children it produces. In sheep breeding, sheep udder traits are correlated with growth rates of weaned lambs.^2^ Optimal udder and teat traits in ewes can significantly improve lamb survival and growth.^3^ The optimal udder and teat traits in ewes significantly improve lamb survival and growth.^3^ In sheep, polydactylous rams and ewes generally have 2–6 teats with a small anterior and large posterior distribution. Lactation performance studies have shown that some polydactyls can lactate normally.^4^ Some studies have found that some polydactyls can lactate normally.^5^ A study on removing multiple teats in sheep found that lactation yield was significantly reduced, but there was no difference in lactation yield between 2 and 4 teat.^6^ The results showed a significant reduction in milk production, but no difference in milk production between 2 and 4 teats.^6^ The localization of functional genes for multiple teats in sheep will facilitate the pace of scientific research on new lines of multiple teats in sheep and goats, as well as breeding selection. In sheep, teats are a highly heritable trait, and the development of extra teats is a complex multigenic trait.^7^ In pig production, teat number influences growth productivity.^7^ And teat number of pigs can be improved by selective breeding.^8^ This trait can be utilized to increase the number of teats in sheep to enhance the milk production performance of sheep.

Mammalian teats can be classified into functional and nonfunctional teats. Zhao et al. found that *LHFP, DPYSL2* and *TDP-43* genes were associated with the formation of teat number in Hu sheep through a GWAS study of HD chip data from populations with different teat counts.^8^ Peng et al. conducted GWAS analysis of HD SNP chip data from sheep and screened out *BBX, CD47* and *CASK* genes associated with the formation of paramecium in sheep, and found that Wnt, Oxt ytcoin, MAPK and axon guidance pathway were important signaling pathways affecting paramecium formation in sheep.^4^ Laura et al. showed that sheep redundant teats have similar immune cells and similar structures such as lymphoid follicles as normal teats, but there is a significant increase in the length and throughput diameter of the redundant teats compared to normal teats.^7^ In a study of commercial sows, Audrey et al. found that elevated numbers of functional teats increased the number of weaned piglets.^9^ The genes associated with teat number are different in different animals. BOVO et al. found that *NUDT* and *NAR6A1* genes were associated with teat number in African rabbits through GWAS study and Fst analysis of 50K chip data in African rabbits, which differed from the candidate genes associated with teat number in pigs.^10^ GWAS analysis of 60K SNP chip data from three pig species by Tang et al. found that the *VRTN* and *KDM6B* genes were closely related to teat number formation in pigs.^11^ Yang et al. GWAS analysis of 50K chip data from pigs revealed that *VRTN* and *ABCD4* genes influenced teat number formation in pigs.^12^

The extreme imbalance between high fertility and low lactation in Hu sheep results in lambs with light birth quality, low survival rate and growth retardation, which becomes a bottleneck restricting the breed’s ability to maximize farming profitability, and death in the form of lambs is the greatest loss to sheep farming enterprises. Currently, most studies on mammalian teat number use GWAS and other analytical methods. In this study, we analyzed the selection signals of HD chip data of 157 Hu sheep from a multiple teats population and a two-teat population in order to screen out candidate genes related to the formation of multiple teat number in Hu sheep through different analytical methods in anticipation of providing data references for the selection and breeding of Hu sheep.

## Materials and methods

### Experimental materials

**Figure 1.**
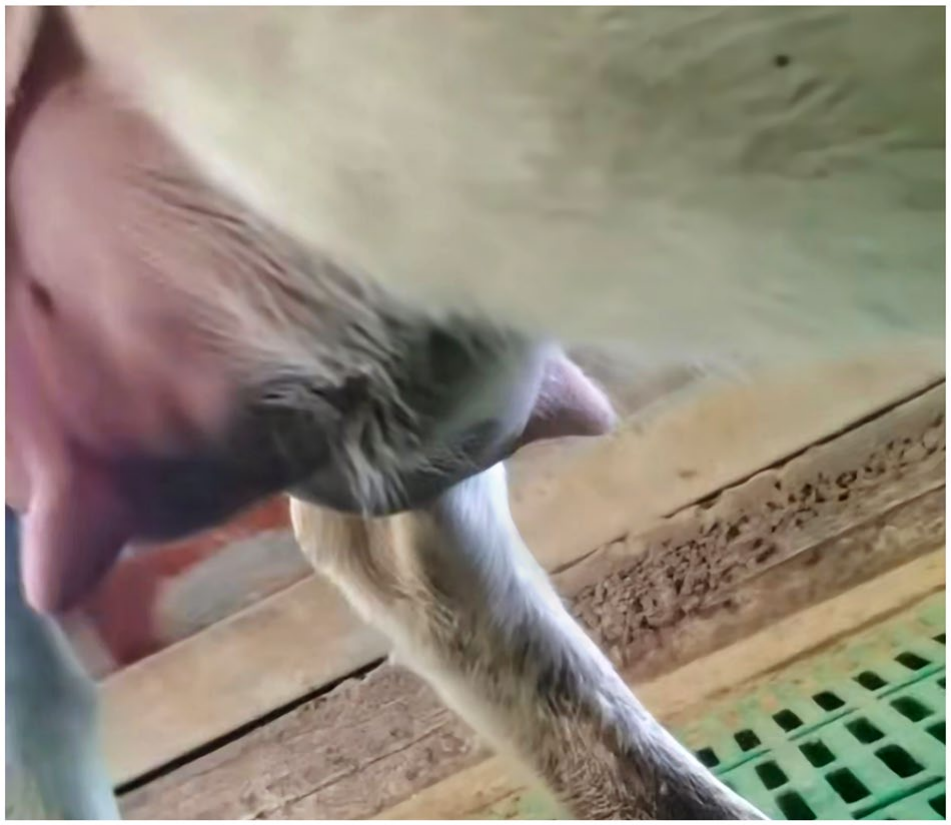
Pictures of normal teats in Hu sheep. Pictures of Hu sheep with two teats.

**Figure 2.**
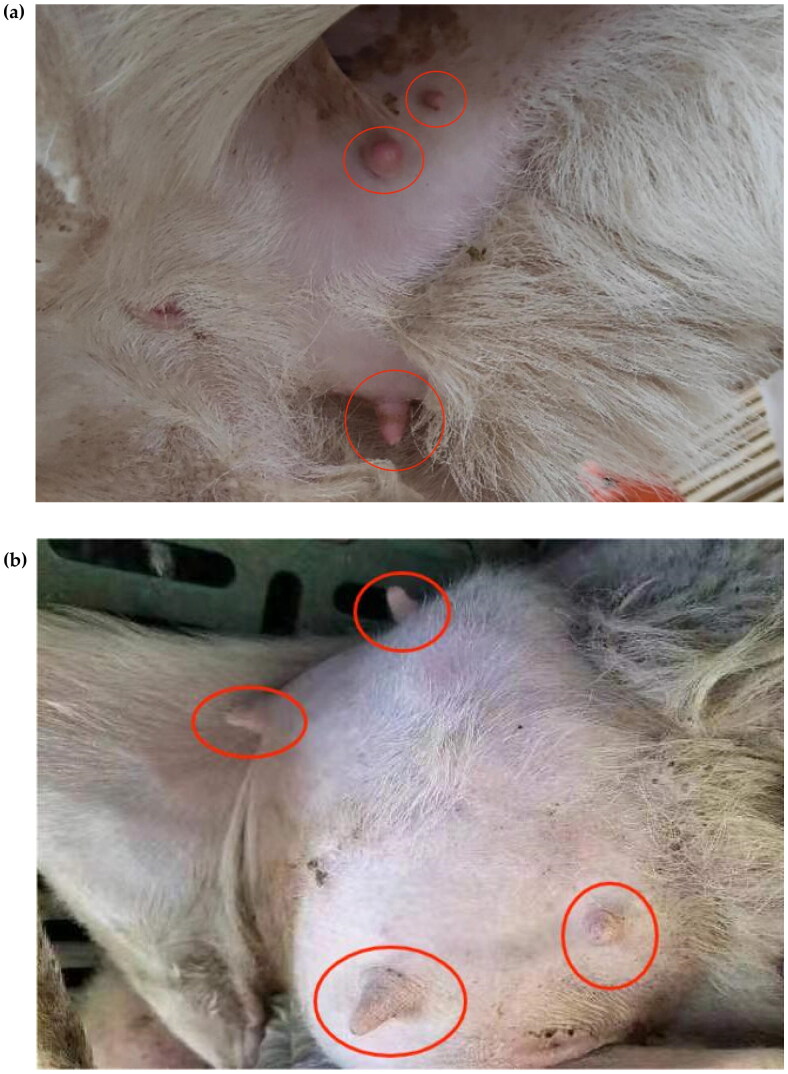
Pictures of Hu sheep with extra teats. (a) The picture shows a photograph of three teats of a lake sheep. (b) The picture shows the four teats of a Hu sheep.

In this study, genome-wide data of 157 Hu sheep from a normal group of 76 two-teaters (Figure 1) and a group of 81 with extra teats were used (Figure 2), which were obtained from https://figshare.com/s/197c20e3229490ccb5d6. Phenotypic data were obtained from https://figshare.com/s/0b326ab8632bbd1c82d6.^8^ The data set was filtered by the following criteria: (i) absence of SNPs for chromosomal and physical location; (ii) SNPs >0.1 for genotypic deletions; (iii) SNPs <0.01 for minor allele frequencies; (iv) individuals with genotyping rates <90%; (v) *p*-values for Fisher’s exact test Hardy–Weinberg equilibrium <0.00001.

### Test methods

Different biological analysis tools have different results, but no single method can completely detect all biological results in genome-wide selective scanning analysis.^13^ In order to capture genomic selection signals more accurately, multiple methods are often required.^13^ For this purpose, we chose four complementary statistical analysis tests: Fst (fixation index), xp-EHH (cross population extended haplotype homozygosity), iHS (integrated haplotype homozygosity score) and Pi (Population nucleic acid diversity). In this study, Fst, xp-EHH were analyzed between the two-teat and multi-teat populations, and Pi, iHS were analyzed separately for the multi-teat population.

#### F_st_ analysis

Calculation of *F_st_* (Fixation Index) for two-teat and multi-teat groups using VCFTOOLS.^14^ It is a measure of genetic differences between populations and is used to determine the degree of genetic differentiation between populations; higher *F_st_* values mean greater genetic differences between populations and vice versa. *F_st_* analysis allows for the study of genetic diversity between populations or subspecies. We used VCFTOOLS for *F_st_* analysis, set the sliding window to 50,000 bp and the step size to 12,500 bp. The formula for *F_st_* analysis is:

$$F_{\text{st}}=\frac{\text{MSP}-\text{MSG}}{\text{MSP}+(N_{C}-1)\text{MSG}}$$

MSG is the mean square of error for detected within-population loci, *MSP* is the mean squared deviation of loci between tested populations, and *N_C_* denotes the corrected mean between overall sample sizes.

#### PI analysis

The multiple teat population of Hu sheep was analyzed for population nucleic acid diversity (Pi) using VCFTOOLS. We set the sliding window of the Pi analysis to 500 kb set the step size to 50 kb, and the SNP loci with 5% of the final Pi value were considered as significant candidates^15^ that were used to analyze polymorphism in the multiple teat groups in Hu sheep.

#### xpEHH analysis

We used rehh Hu sheep genomic data to do heterozygous extended haplotype purity tests (*xpEHH*),^16^ Rehh (R package for detecting recent positive selection using extended haplotype homozygosity) is a statistical tool for detecting selection signals based on extended haplotype homozygosity (*EHH*). It can be used to identify positive selection events occurring in genetic variants in the human genome.

The *xpEHH* statistic was calculated by comparing the two-teat group to the multiple-teat group with the formula:

$$\text{xpEHH}=\frac{\text{unxpEHH}-\text{mean}(\text{unxpEHH})}{\text{sd}(\text{unxpEHH})}$$

where *unxpEHH* is set:

$${\text{unxpEHH}}_{\text{scores}}=\text{In}(\frac{{\text{iES}}_{\text{pop}1}}{{\text{iES}}_{\text{pop}2}})$$

*iES_pop1_* integrates *EHH* genetic distance statistics, and *iES_pop2_* integrates genetic distances for *EHH* statistics for multi-teat populations.

#### iHS analysis

Separate polypodium clusters for integrated haplotype purity scoring (*iHS*),^17^ a single marker locus was used to replace the core haplotypes in the *EHH* statistic, defining them as core loci. And their ratios were calculated to select the signal detection statistic used to complement the *F_st_* analysis, calculated as:

$$\text{iHS}=\frac{\text{uniHS}-\text{mean}(\text{uniHS}|p_{s})}{\text{sd}(\text{uniHS}|p_{s})}$$

where *uniHS* is set:

$$\text{uniHS}=\text{In}(\frac{{\text{iHH}}_{A}}{{\text{iHH}}_{D}})$$

*IHH* is the integration of genetic distances of *EHH* (integrated *EHH*), a represents the ancestral (progenitor) allele, and *D* represents the new mutant (derived) allele.

### SNP annotation and analysis

For each scan the results were visualized using-R. We took the first 5% of SNP sites for *F_st_*, *xpEHH*, and *iHS* values, and only the last 5% of SNP sites for Pi analysis were taken as positively selected sites to identify the selected regions in the genome, and all the obtained SNPs were annotated based on Sheep Oar_v4.0 (https://www.sheephapmap.org/). Genes were functionally analyzed with reference to the NCBI database (http://www.ncbi.nlm.nih.gov/gene), and visual intersection analysis was performed on individual analytical annotations using Draw Venn Diagram (Draw Venn Diagram (ugent.be)), using the DAVID tool (http://david.Abcc.ncifcrf.gov/) for gene ontology database (GO, http://geneontology.org)^18^ and Kyoto Encyclopedia of Genes and Genomes (KEGG, Kyoto Encyclopedia of Genes and Genomes)^19^ analysis. Related candidate genes for protein interactions analyzed on the STRING website (https://cn.string-db.org/).

## Results

### F_st_ analysis results

**Figure 3.**
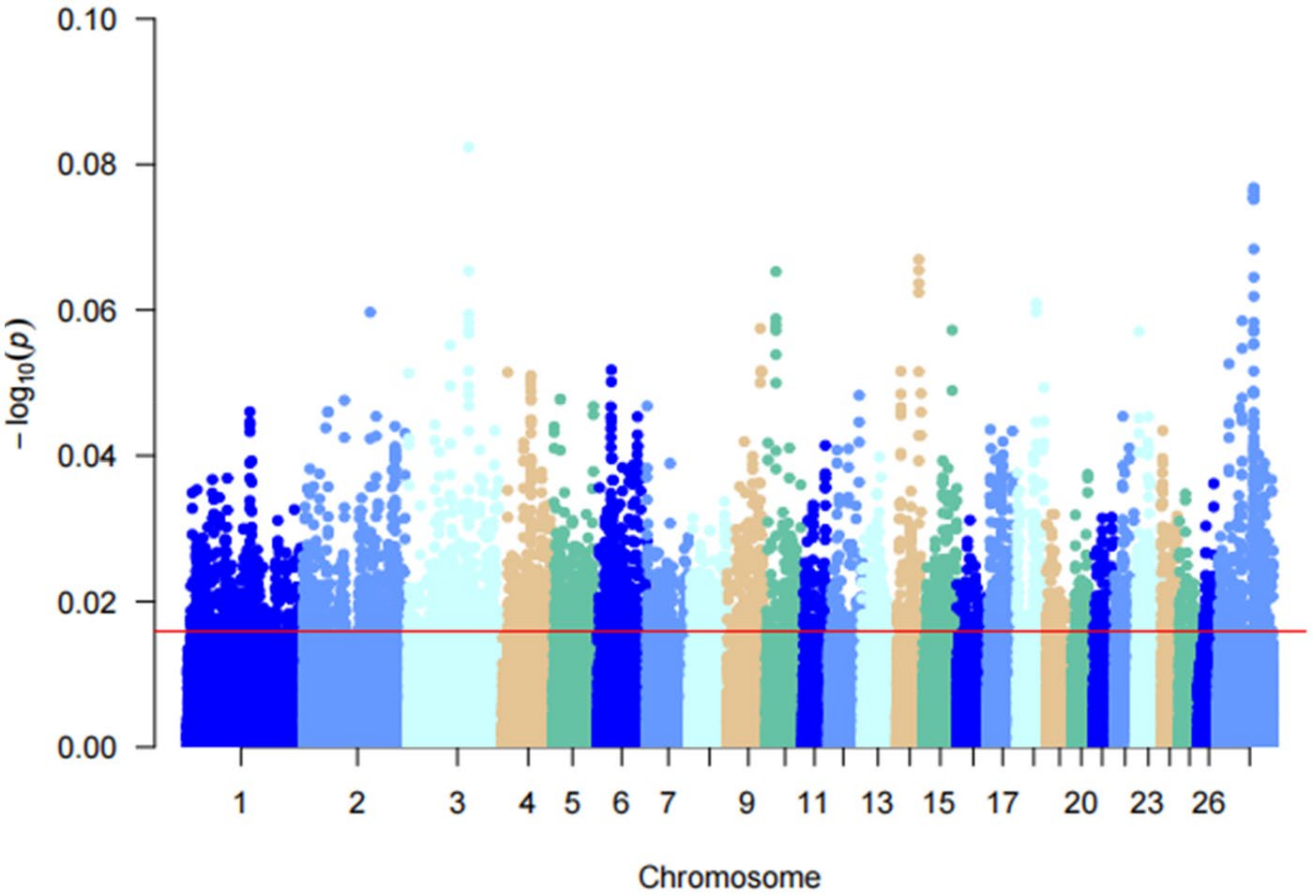
Manhattan plot of *F_st_* analysis. The different colors in the figure represent the different chromosomes 1–27, and the thin red line in the figure represents the value of *F_st_* = 0.115419, which is the value of the top 5% of the *F_st_* analysis.

Multiple teats are a special phenotypic feature of Hu sheep compared to two teats. Multiple teats population favors the survival of lambs with multiple births. Using PLINK 19.0 software, *F_st_* analysis was performed on the two-teat and multi-teat populations of Hu sheep, and the top 5% of *F_st_* values. And the regions with *F_st_* values >0.012 were considered as candidate regions for the multi-teat population and its significant loci were mainly distributed on chromosomes 3, 14, 2 and 8. A total of 10,278 loci were selected, and gene annotation was performed on the selected regions (Figure 3), resulting in 2088 genes.

### Results of Pi analyses

**Figure 4.**
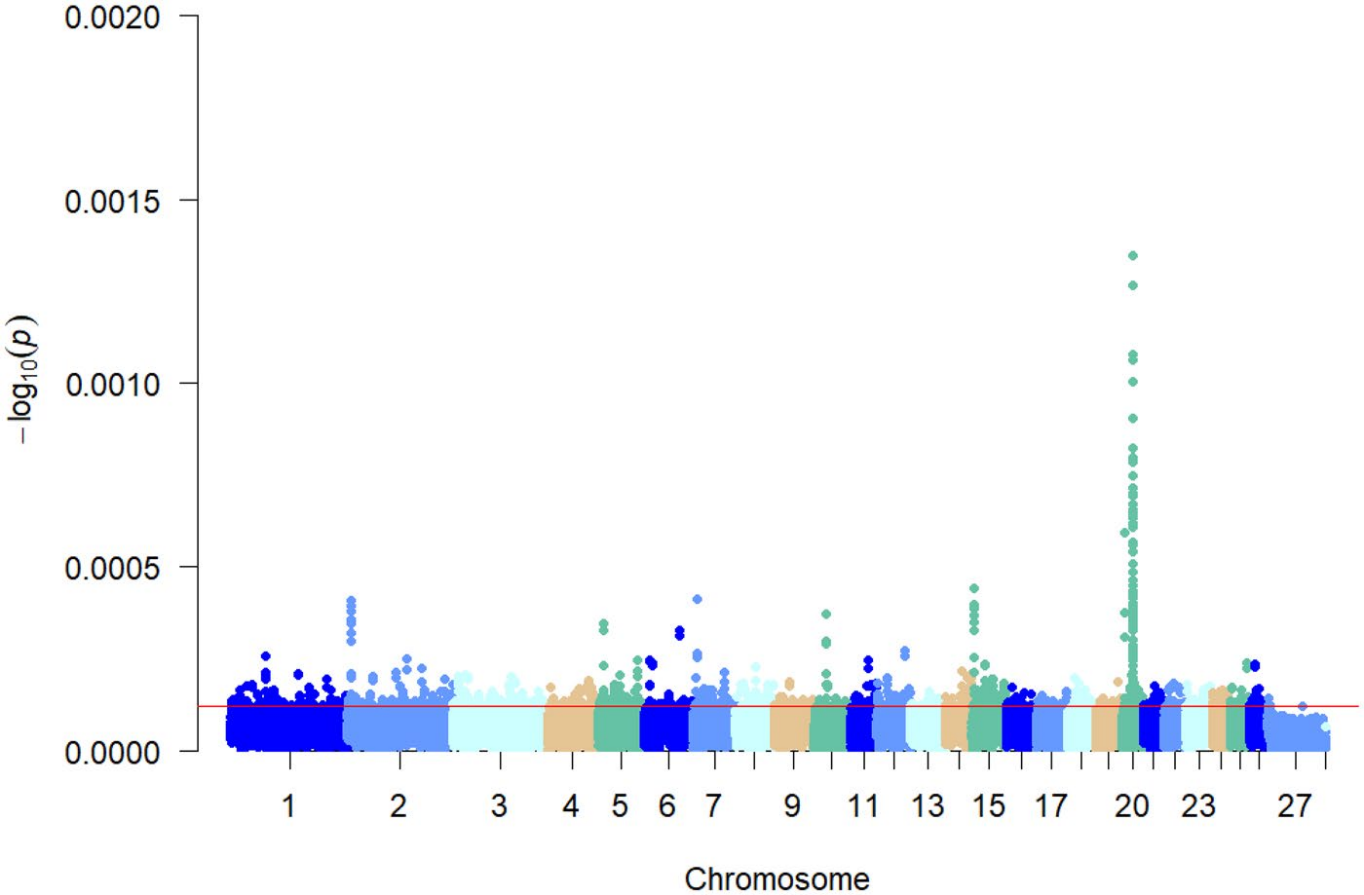
Manhattan plot of Pi analysis results. This figure shows the pi analyzed values for a multiple teats population of Hu sheep. Different colors in the figure represent different chromosomes and the thin red line represents the top 5% threshold line.

Nucleic acid polymorphisms were analyzed in the multi-teat population of Hu sheep, and the top 5% of the Pi values, with a Pi value >0.000106168, were considered as the selected regions, and 5140 SNP loci were selected (Figure 4), and annotation analyses were performed on the selected regions, and 3,557 genes were obtained.

### Results of xpEHH analyses

**Figure 5.**
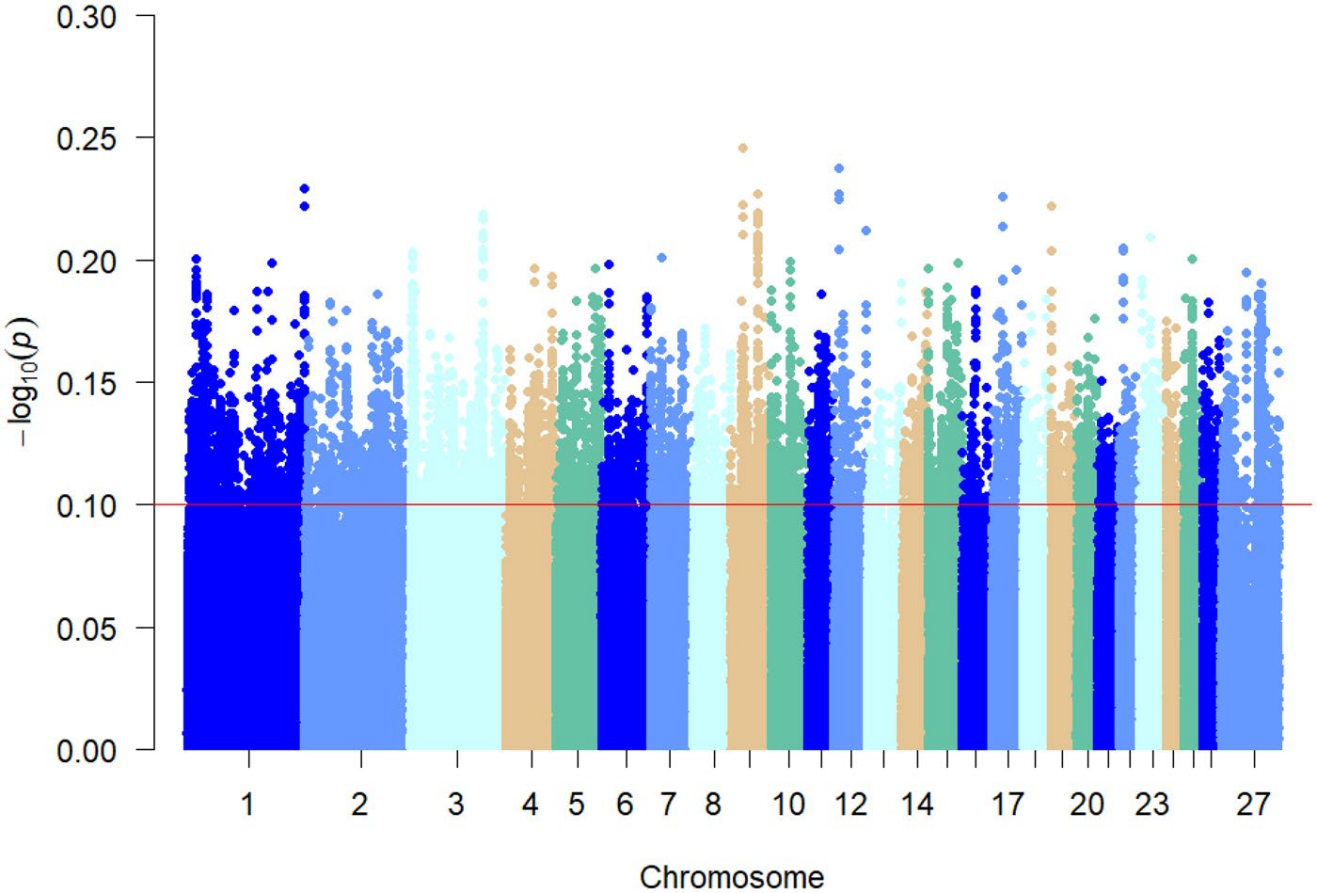
Manhattan plot of *xpEHH* analysis results. Different chromosomes are represented by different colors, red line in the figure represent candidate regions selected for *xpEHH* analysis.

The *xpEHH* analyses were performed using VCFTOOLS on the multi-teat and two-teat populations of Hu sheep by comparing the extended haplotype purity differences between the two populations, with the two-teat population serving as the reference population and the multi-teat population serving as the test population for the selection signals analyses. As shown in Figure 5, the top 5% of the absolute value of the *xpEHH* analysis results were selected as the loci in the multiple teat population, and 1779 SNP loci were found in the multi-teat population, and 1779 genes were obtained after annotation analysis of the relevant loci.

### Results of iHS analyses

**Figure 6.**
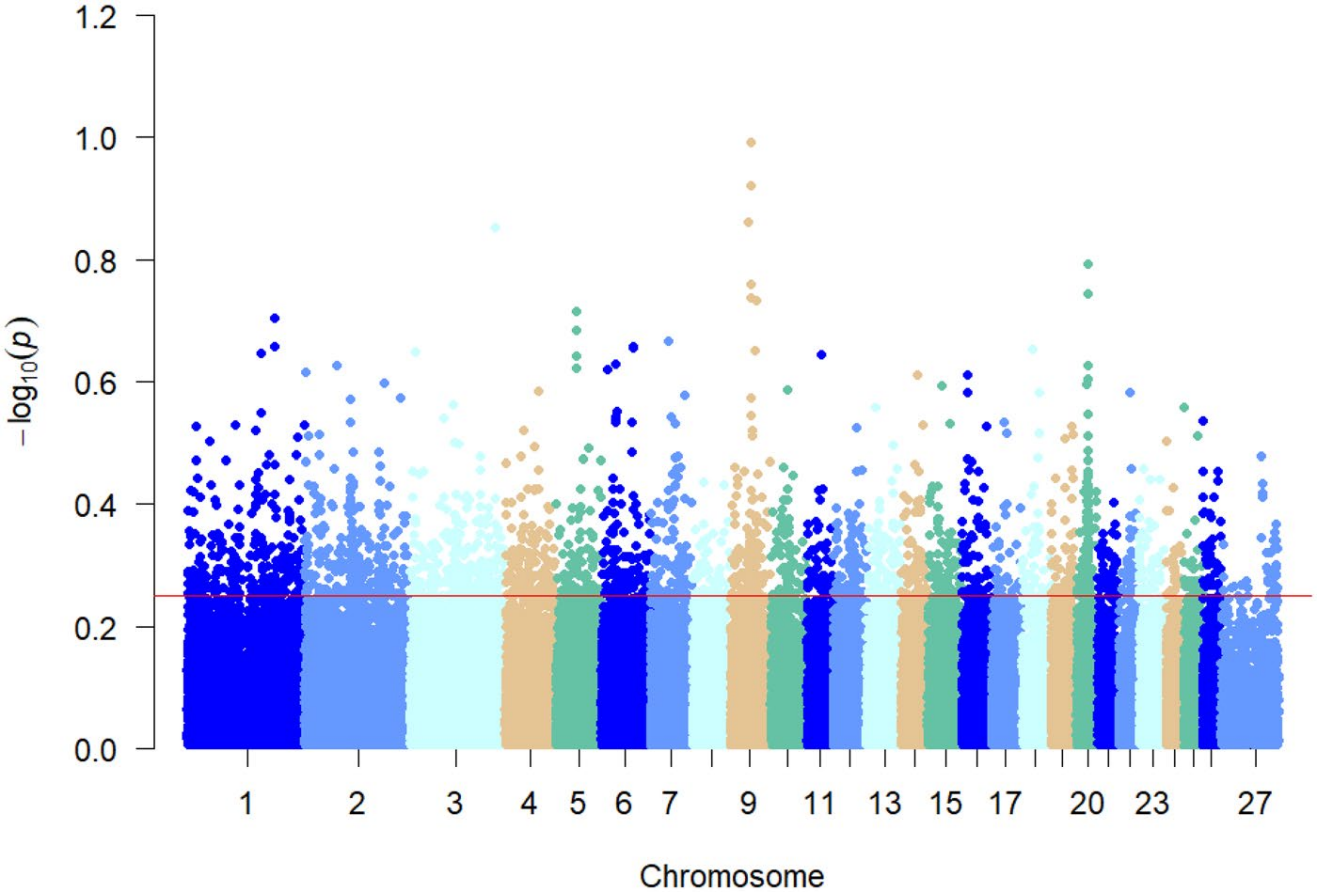
*iHS* Analysis of the manhattan diagram notes. The red line in the graph represents the top 5% threshold line.

*iHS* detects strong selection signals in favor of alleles that have not yet reached fixation.^17^ In this study, *iHS* analysis was performed individually on the Hu sheep multiple teat population, as shown in Figure 6. The top 5% of the absolute value of the *iHS* results were considered as the region of selection, and 23,558 were the selected loci. Chromosomes 1, 7, 10, 20, and 21 showed peaks indicating loci on these chromosomes that were partially not yet fixed. Annotation analysis of the significant candidate loci yielded 3968 genes.

### Veen analysis and gene enrichment

The sheep 4.0 genome was used as a reference to annotate the candidate-selected regions analyzed by *F_st_*, Pi, *xpEHH* and *iHS*, as shown in Figure 7(a), and 142 genes were overlapped (Table 1). For further functional analysis of the overlapped genes, we used DAVID online software to analyze the GO and KEGG pathways of the 142 genes, as shown in Figure 7(b,c).

Figure 7.Veen diagram and enrichment analysis. Figure 5(a) Shows the common genes annotated from the candidate SNP loci for the four analyses. The 0.05*F_ST_* in blue in the figure represents the genes annotated for the top 5% of SNPs loci analyzed by *F_st_*. The light red part 0.05 *iHS* analyzed for the first 5% of SNPs loci, 0.05 PI in green in the figure represents the genes annotated from the first 5% of SNPs loci analyzed by Pi, and 0.05 *xpEHH* in yellow in the figure represents the genes annotated from the first 5% of SNPs loci analyzed by *xpEHH*. (b) Shows the bubble plot of GO enrichment analysis of candidate genes. (c) Shows the bubble plot of KEGG enrichment analysis of candidate genes.

**Figure:**
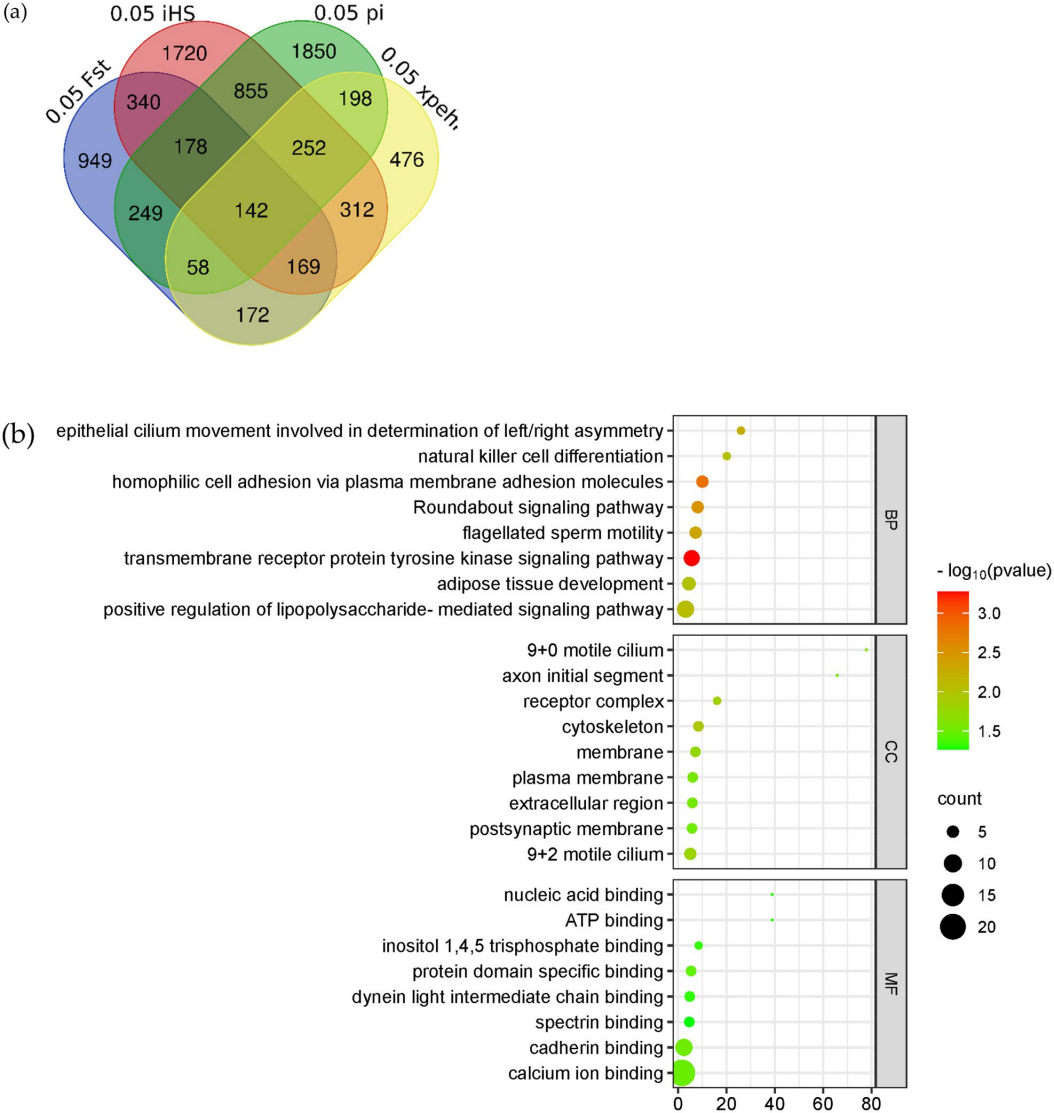

**Figure:**
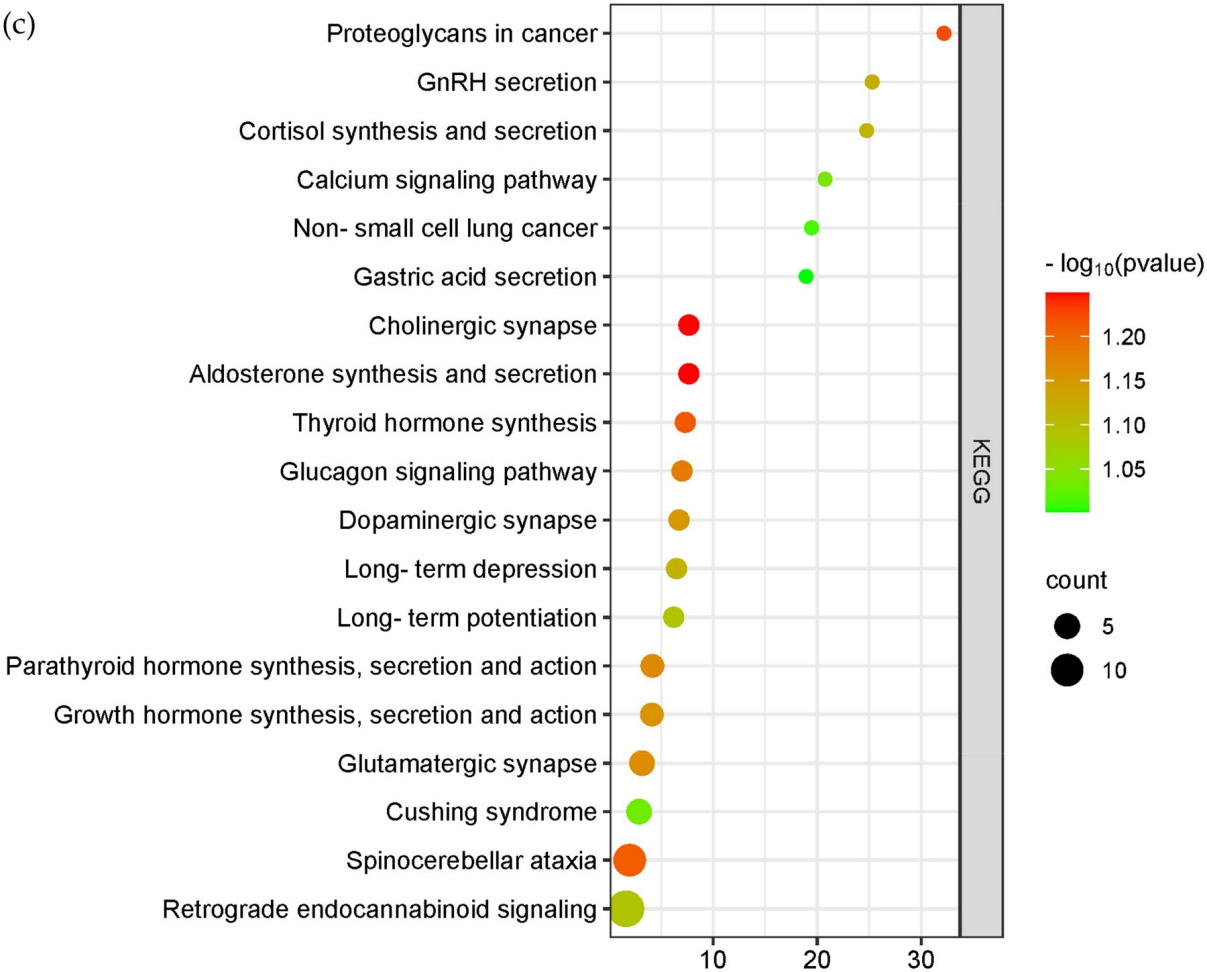

**Table 1.** Candidate gene correlation analysis values. The genes presented in the table are candidate genes common to the four selection signal analyses in the text.

Candidate gene analysis values					
*F_st_*	*xpEHH*	Pi	*iHS*	Chrom	Gene name
0.0125134	0.0969546	0.000137341	0.169807	10	*FARP1*
0.0141406	0.102782	0.000138787	0.155412	5	*MYO9B*
0.0136369	0.0971815	0.000129846	0.272623	3	*ATXN10*
0.0198374	0.0892936	0.000108544	0.141152	4	*DNAH11*
0.0140788	0.13531	0.000126995	0.096153	3	*LONRF2*
0.0180752	0.114316	0.000117083	0.201886	1	*IL23R*
0.0143596	0.0905919	0.000113971	0.223994	6	*PPARGC1A*
0.0128181	0.0957009	0.000112079	0.326865	2	*ASTN2*
0.0211912	0.166071	0.000116878	0.449407	9	*CSMD3*
0.0140285	0.0998928	0.000128737	0.203701	23	*DLGAP1*
0.0359107	0.106669	0.000111978	0.233394	2	***LRP1B***
0.017715	0.0904149	0.000111504	0.244635	1	*NEGR1*
0.0167893	0.0979947	0.000121609	0.238596	3	*TRHDE*
0.0250087	0.140692	0.000116135	0.287968	3	*NELL2*
0.028746	0.116311	0.000130173	0.210642	23	*LAMA3*
0.0300968	0.116607	0.000126524	0.115656	4	*DPP6*
0.0121405	0.125584	0.000124213	0.202213	25	*GRID1*
0.0159519	0.0855406	0.000106478	0.174637	3	*COMMD1*
0.0126131	0.0807562	0.00015858	0.211059	11	*ABCA6*
0.0198632	0.123574	0.000109622	0.0873149	19	***PTPRG***
0.0226703	0.106955	0.000132225	0.205564	1	*FILIP1L*
0.0127494	0.0833948	0.000114888	0.280595	9	*KCNQ3*
0.020226	0.0984031	0.000107705	0.292845	22	*ATRNL1*
0.0200469	0.105022	0.0001104	0.158133	8	*UST*
0.0202424	0.152509	0.000112369	0.210909	2	*SH3GL2*
0.0287782	0.15284	0.000108731	0.278738	1	***DOCK7***
0.0189957	0.138567	0.000127119	0.286006	1	*ROR1*
0.0247168	0.14545	0.000110012	0.179432	3	*AFF3*
0.0236198	0.0980806	0.00010693	0.228344	2	*ERBB4*
0.0208061	0.179315	0.000106989	0.107739	7	*EPB41L4A*
0.0133874	0.105651	0.000161158	0.000161158	22	*PTPRE*
0.0167454	0.0864918	0.00010626	0.133672	14	*PRSS54*
0.0144944	0.102314	0.000119282	0.254217	2	*SPAG16*
0.0159174	0.0857668	0.000118954	0.315966	2	*ZRANB3*
0.0247274	0.101588	0.000116345	0.215574	19	*CNTN4*
0.0234435	0.122773	0.00010872	0.19239	4	*SND1*
0.0203703	0.0994272	0.000108947	0.11489	12	*ENAH*
0.016176	0.0901188	0.000181069	0.17421	6	*ANK2*
0.0136707	0.0977661	0.000126762	0.184579	22	*CPN1*
0.0116329	0.0987518	0.000124808	0.280894	16	*SLIT3*
0.01365	0.0875218	0.000109289	0.211522	1	*ROBO1*
0.0153057	0.111301	0.000108326	0.202917	19	*ITPR1*
0.0176753	0.149455	0.000109367	0.150048	19	*IL17RB*
0.0127748	0.103659	0.000179457	0.152553	11	*FTSJ3*
0.0306481	0.0861801	0.000111007	0.24795	3	*ITPR2*
0.0488301	0.196328	0.000114648	0.494689	4	*JAZF1*
0.0119866	0.107459	0.000107881	0.101285	4	*PPP1R3A*
0.0161019	0.0924775	0.000114488	0.226897	5	*EDIL3*
0.0138765	0.145718	0.000151757	0.0999466	15	*TCN1*
0.0273828	0.229155	0.000112944	0.202219	1	*KCNH8*
0.0247217	0.132741	0.000110542	0.409258	3	*DIP2B*
0.0266611	0.111026	0.000117511	0.129541	9	*COLEC10*
0.0167149	0.108635	0.000122642	0.115138	6	*TBC1D1*
0.0187447	0.117845	0.000114142	0.21125	24	*SHISA9*
0.0118631	0.0846617	0.000119908	0.138795	11	*MSI2*
0.0514379	0.14704	0.000117351	0.166087	4	*DYNC1I1*
0.0142223	0.114591	0.000113487	0.0927003	11	*TNRC6C*
0.0184914	0.132091	0.000112948	0.247652	19	*SYNPR*
0.0157646	0.0994011	0.000116483	0.134906	1	*ATP13A5*
0.0304602	0.123526	0.000108335	0.305165	19	*RBMS3*
0.0131376	0.0970959	0.000125877	0.160061	21	*ME3*
0.0151188	0.138775	0.000111976	0.147204	26	*ZMAT4*
0.0187297	0.089567	0.000106193	0.198316	11	*WNT9B*
0.0155832	0.103169	0.000272938	0.456257	12	*CENPF*
0.017966	0.136204	0.000120394	0.124856	22	*TACC2*
0.0185585	0.116945	0.000114885	0.142182	4	*IGF2BP3*
0.016965	0.111521	0.000114206	0.161755	8	*UTRN*
0.0353733	0.131086	0.000127401	0.214695	1	*AGBL4*
0.0205176	0.126865	0.000108182	0.185674	24	*RBFOX1*
0.0377021	0.108956	0.000159571	0.143001	24	*ZNF75A*
0.0128484	0.119562	0.000107236	0.153584	3	***TMEM117***
0.0127333	0.089255	0.000107953	0.193532	11	*WNT3*
0.0247217	0.0925592	0.000110542	0.137656	3	*ATF1*
0.0125574	0.0873669	0.000129163	0.262501	18	*SLC25A21*
0.0182351	0.100485	0.000139822	0.107074	2	*AKNA*
0.0122888	0.080822	0.000131315	0.151024	21	*NTM*
0.0125437	0.0906824	0.00011277	0.176868	17	*RIMBP2*
0.0153819	0.134034	0.000126995	0.1129	3	*CHST10*
0.0193773	0.0987052	0.000126995	0.245499	19	*FHIT*
0.0184189	0.136008	0.000107743	0.130126	9	*TOX*
0.0182553	0.119871	0.000109367	0.15323	19	*CHDH*
0.0229908	0.130526	0.000115598	0.239359	22	*PCDH15*
0.0120501	0.0833012	0.000116726	0.350292	19	*ERC2*
0.0238652	0.0898589	0.000107236	0.16631	9	*RIMS1*
0.0171986	0.104021	0.000114169	0.173474	19	*NEK10*
0.0127695	0.212083	0.000159236	0.334631	12	*PTPRC*
0.0159637	0.104666	0.000108702	0.204044	8	*SASH1*
0.0226703	0.1191	0.000132225	0.205564	1	*CMSS1*
0.0204131	0.0989284	0.000144231	0.103201	21	*GTF2H1*
0.0126131	0.0845532	0.000172712	0.284135	11	*ABCA10*
0.014493	0.121616	0.000109893	0.0916305	6	*LIAS*
0.0307172	0.0871735	0.000114002	0.200442	4	*POU6F2*
0.0161839	0.100632	0.000118706	0.286596	21	*OPCML*
0.0291673	0.0810811	0.000118184	0.127225	2	*ATF2*
0.0143623	0.0855181	0.000119798	0.160472	2	*ACVR1*
0.0166242	0.110417	0.000130349	0.176624	21	*SYTL2*
0.0151202	0.116888	0.000109073	0.169802	13	*CELF2*
0.014949	0.101819	0.000108395	0.212098	5	*SGCD*
0.0286155	0.133724	0.000144605	0.227797	1	*PHTF1*
0.0126866	0.0829231	0.000111916	0.179042	4	*ICA1*
0.0201986	0.0844209	0.000128055	0.113099	22	*LOXL4*
0.0326285	0.138967	0.000121828	0.215216	11	*NLRP1*
0.0221557	0.119844	0.000122121	0.232353	7	*NRXN3*
0.0186338	0.0796293	0.000133901	0.135295	2	*BSPRY*
0.011987	0.123649	0.000117114	0.360086	13	*MACROD2*
0.020053	0.10266	0.000135996	0.109809	17	*DCHS2*
0.0158716	0.133275	0.000131231	0.165556	16	*RAB3C*
0.0336139	0.130163	0.000107625	0.294293	14	*CDH13*
0.0128935	0.108603	0.000119741	0.339256	1	*DAB1*
0.014642	0.0850342	0.000129501	0.2363	3	*ALK*
0.0266508	0.12241	0.000112674	0.120674	3	*SLC2A3*
0.0230233	0.123364	0.000144605	0.227797	1	*MAGI3*
0.0166619	0.115759	0.000115855	0.166924	4	*VPS41*
0.0245185	0.131403	0.000180443	0.106776	23	*ZNF407*
0.0234623	0.111376	0.000107665	0.300443	5	*FSTL4*
0.0223086	0.0802089	0.000108544	0.170743	4	*CDCA7L*
0.0185054	0.0893213	0.000145745	0.200327	25	*WDFY4*
0.0151169	0.116915	0.000111692	0.389165	6	*COL25A1*
0.0202696	0.114126	0.00011648	0.194774	2	*THSD7B*
0.0157284	0.0915644	0.000110284	0.160306	6	*PGM2*
0.0175023	0.143651	0.000115178	0.242156	13	*SLCO4A1*
0.0178244	0.095394	0.000123052	0.206526	22	*SORCS1*
0.0270416	0.122419	0.000116006	0.328892	21	*DLG2*
0.0144588	0.113309	0.000126162	0.484322	2	*NCKAP5*
0.0169597	0.143806	0.000106533	0.138327	2	*SCARA5*
0.0124323	0.112974	0.000124546	0.164256	16	*LIFR*
0.0166625	0.0927614	0.00011998	0.00011998	17	*TTC28*
0.0133908	0.107662	0.000107254	0.228132	2	*GALNTL6*
0.0136874	0.118105	0.000126995	0.152868	11	*ASIC2*
0.0144645	0.0978911	0.000137953	0.148899	15	*LDLRAD3*
0.0165791	0.0852525	0.000123718	0.232836	21	*FAT3*
0.0191899	0.110809	0.000125693	0.272377	4	*CHRM2*
0.0208294	0.141616	0.000166036	0.177876	25	*CDH23*
0.0129701	0.140614	0.000113007	0.230124	5	*CHSY3*
0.0231262	0.130589	0.000107305	0.234443	3	*CTNNA2*
0.0171521	0.139332	0.000113059	0.215997	2	*PARD3B*
0.0159097	0.091068	0.000120543	0.232896	11	*CEP112*
0.0208248	0.0861008	0.000118445	0.25845	11	*PRKCA*
0.0167998	0.097787	0.000153543	0.526849	16	*DNAH5*
0.0362153	0.167257	0.000116439	0.0972408	26	*LRRC3B*
0.0164443	0.124258	0.000112898	0.118093	9	*FAM49B*
0.0184089	0.0908601	0.000126343	0.225265	1	*CFAP45*

The bolded font in this table represents important candidate genes.

**Figure 8.**
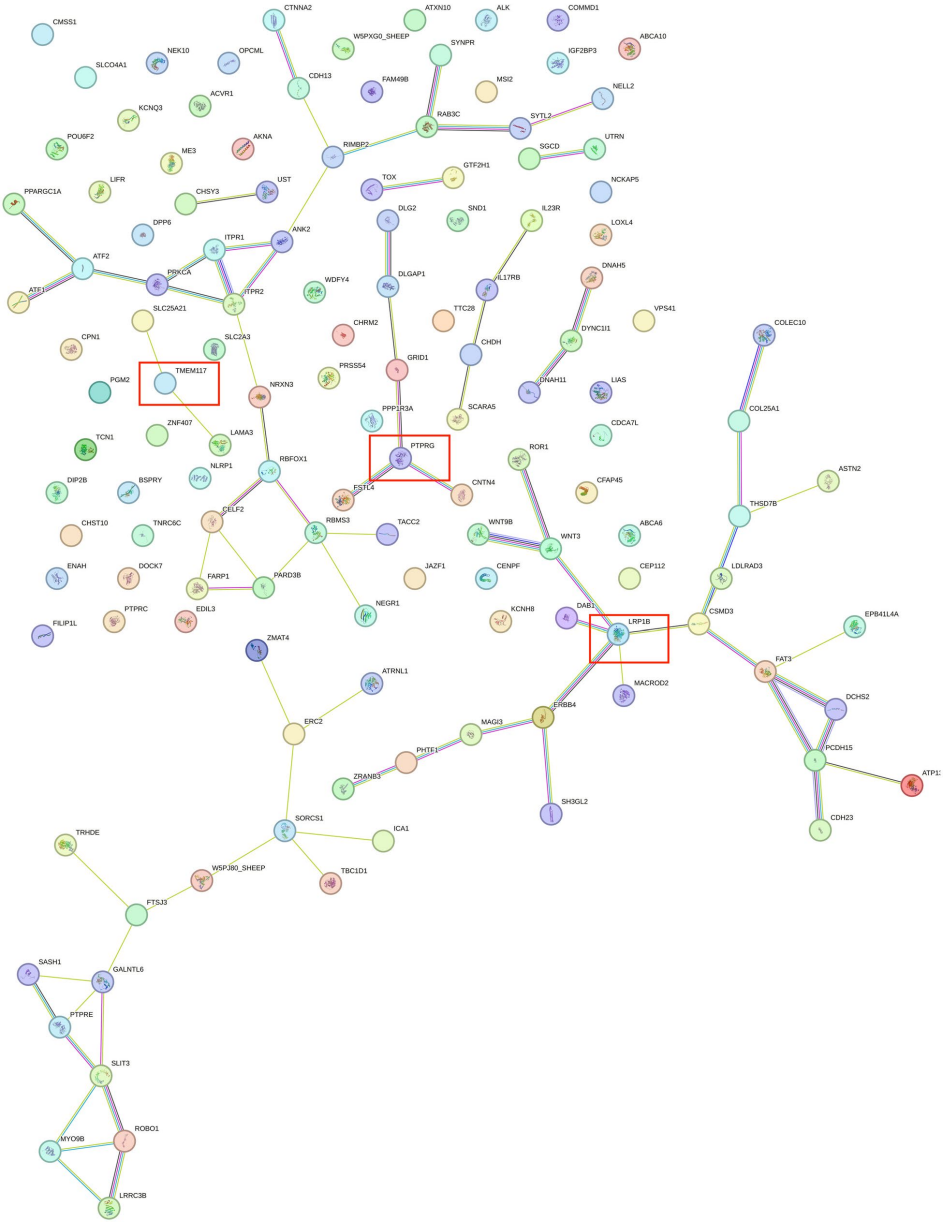
Related candidate genes protein interaction diagram. The genes identified by the red boxes in the figure are thought to be strongly correlated with the formation of the polydactyl trait in Hu sheep.

Peng et al. showed that paraplasia in sheep may be mediated by regulating cell proliferation, and that the selective signaling pathway of its occurrence is involved in breast cancer development.^6^ In this study, calcium binding pathway (GO:0005509) was shown and *NELL2, CDH23, PCDH15, CDH13, SLIT3, FAT3, EDIL3, LRP1B, FSTL4* genes were shown. In order to screen out the candidate genes among the further candidate genes related to polydactyly in Hu sheep, we carried out gene interactions analyses on the related genes. As shown in Figure 8, the *PTPRG, TMEM117* and *LRP1B* genes were shown to be associated with multiple genes.

## Discussion

Detection of genomic regions under positive selection specific to a population is essential to reveal the genetic basis of variation in localized adaptive traits.^20^ In this study, we genotyped genomic data between a multiple teat population and a two teats population of Hu sheep for *F_st_* analysis. Fixation indices analysis is a measure of whether the actual frequency of genotypes in a population deviates from the theoretical proportion of genetic equilibrium (Harwin’s equilibrium). The genetic structure did not show a large genetic differentiation between the multi-teat population and the two-teat population of Hu sheep. In order to reduce the interfering factors of *F_st_* analysis and increase the accuracy of selection signal detection. Screened the selected genes for the two populations of Hu sheep based on the theory of linkage disequilibrium as well as the extended haplotype purity test for the positive characteristics of the haplotypes. Analyzed the allele frequency analysis between the multi-teat and two-teat populations of Hu sheep using xp-EHH analysis as well as the pure haplotypes of multi-teat populations of Hu sheep for *iHS* analyses were complemented. The correlation analysis of its haplotypes revealed that the *xpEHH* analysis of its haplotypes revealed that the candidate *xpEHH* values ranged from 0.08 to 0.25 and the highest *xpEHH* value was found on chromosome 9. The same *iHS* analysis showed that the locus associated with multi-teat was fixed on chromosome 9. Its nucleotide diversity showed that loci with higher diversity were mainly located on chromosome 9. In addition, Pi analysis was used to screen the genomic selection signals of the multi-teat population of the Hu sheep. The highest Pi value was 0.014, which revealed that the subpopulation diversity of the polyposis population of the Hu sheep was not high. And based on the above analyses, we considered that the heritability of the multi-teat population of the Hu sheep was high.

In order to further identify the candidate genes related to multi-teat formation in Hu sheep, we annotated the overlapping regions of the four analysis methods, and among the 143 candidate genes screened for association with multi-teat. Based on the results of the gene interactions map we identified the mammary gland-related *PTPRG,*^21^ *TMEM117*^22^ and *LRP1B* genes.

*Protein tyrosine phosphatase receptor type G* (*PTPRG*) gene, located on sheep chromosome 19 at position NC_056072.1 (39155807.39930537, complement). This gene has been reported to inhibit mammary epithelial cell motility^23^ and has been associated with growth traits in sheep.^24^ In addition *PTPRG* has been identified as a tumor suppressor gene in breast cancer. The expression of *PTPRG* is reduced during breast carcinogenesis and is particularly low in high malignancy grade breast cancer.^25^ *PTPRG* is involved in the control of *FGFR1* activity and influences the sensitivity of sarcoma cells to *FGFR* kinase inhibitors, and is important for cytostasis in breast cancer.^26^

*The transmembrane protein 117* (*TMEM117*) gene, located on sheep chromosome 3 NC_056056.1 (142302614.142937834, complement), was reported to be associated with posterior udder attachment width in hesitant cows.^22^ Zhu et al.^27^ also conducted a GWAS study and found that the *TMEM117* gene was associated with saturated fatty acid composition in Simmental cows. Xia et al. find gene linked to beef traits^28^ the gene has also been reported to be associated with fatty acid formation in Ethiopian Indigenous Goat. This gene has also been reported to be associated with fatty acid formation in Ethiopian Indigenous Goat.^29^ The gene was also reported to be associated with fatty acid formation in Ethiopian Indigenous Goat.^29^

*LDL receptor related protein 1B* (*LRP1B*) gene, located on chromosome 2 of sheep at position NC_056055.1 (168072439.170268670). was reported to reduce malignant tumor cells in breast cancer cell lines,^30^ in a study by Peng et al. on sheep paramammary glands, *LRP1B* was shown in GWAS analysis and was the significant locus.^6^ This gene has also been reported to be associated with litter size in Greater Montenegrin sheep,^31^ *LRP1B* may contribute to birth weight variability in pigs^32^ and is involved in smooth muscle cell migration in chickens.^33^

Extra teats in Hu sheep have similar immune cell and lymphatic structures as normal teat formation, and it has been shown that part of the extra teats in Hu sheep have the function of milk production, and the screening of individuals with extra teats in Hu sheep has certain benefits for lamb survival and production traits in Hu sheep populations.^7^ In this study, we analyzed the selection signals of excess teats in Hu sheep, hoping to provide a molecular basis for the production as well as screening of excess teats in Hu sheep through genetic aspects. In this study, we found that the genes *PTPRG*, *TMEM117* and *LRP1B* were closely associated with the production of surplus teats in Hu sheep.

In addition, this study identified genes associated with production growth among the relevant candidate genes. Genes such as *DNAH11,*^34^ *NELL2,*^35^ *ANK2,*^36^ *SLIT3,*^37^ *DIP2B,*^38^ *NEK10*,^39^ *GRID1,*^40^ and *PHTF1*^41^ associated with reproductive traits. And *SH3GL2*,^42^ *DOCK7*,^43^ *ENAH*,^44^ *SHISA9*,^45^ *AGBL4*,^46^ *SGCD*,^47^ *COL25A1,*^48^^,^^49^ *DLG2*,^5^ *DOCK7,*^50^ and other genes associated with milk production traits. And *TMEM117,*^22^ *SLC25A21*,^51^ *NCKAP5*,^52^ *PRKCA*,^53^ *ITPR2*,^54^ *NEGR1*,^55^ and other genes, and *ASTN2*,^56^ *GRID1*,^57^ *FILIP1L*,^58^ *ATRNL1*,^59^ and *ERC2*,^60^ which are associated with stress tolerance.

## Conclusions

In this study, candidate genes related to multi-teat in Hu sheep, such as *TMEM117, PTPRG* and *LRP1B* genes, were screened by selective signal analysis of HD chip data. These genes provide data for breed selection of Hu sheep, and also provide reference for the study of other multi-teat.
